# Cancer-associated fibroblast-derived exosomal microRNA-98-5p promotes cisplatin resistance in ovarian cancer by targeting CDKN1A

**DOI:** 10.1186/s12935-019-1051-3

**Published:** 2019-12-21

**Authors:** Hua Guo, Chunfang Ha, Hui Dong, Zhijuan Yang, Yuan Ma, Yonghui Ding

**Affiliations:** 1 Department of Gynecology, General Hospital of Ningxia Medical University, No. 804, Shengli South Street, Xingqing District, Yinchuan, 750004 Ningxia Hui Autonomous Region People’s Republic of China; 2 Scientific Research Equipment Management Center, General Hospital of Ningxia Medical University, Yinchuan, 750004 People’s Republic of China

**Keywords:** Cancer-associated fibroblasts, Exosomes, microRNA-98-5p, CDKN1A, Ovarian cancer, Cisplatin resistance

## Abstract

**Background:**

Ovarian cancer (OC) is a gynecological malignancy with a high mortality. Cisplatin-based treatment is the typical treatment regimen for OC patients; however, it may cause unfavorable resistance. The current study intends to explore the function of cancer-associated fibroblast (CAF)-derived exosomal microRNA-98-5p (miR-98-5p) in cisplatin resistance in OC, and the participation of CDKN1A.

**Methods:**

Bioinformatics analysis was employed in order to obtain cisplatin resistance-related differential genes in OC as well as possible upstream regulatory miRs. After gain- and loss-of-function assays in OC cells, RT-qPCR and western blot analysis were employed to measure CDKN1A and miR-98-5p expression. Dual luciferase reporter assay was applied to verify the targeting relationship between miR-98-5p and CDKN1A. CAFs were treated with miR-98-5p inhibitor, and then exosomes were isolated and co-cultured with OC cells. CCK-8, colony formation and flow cytometry assays were conducted to assess cell proliferation, cell colony formation, cell cycle distribution and cell apoptosis, respectively. At last, xenograft tumor in nude mice was carried out to test whether exosomal miR-98-5p could affect cisplatin resistance in OC in vivo.

**Results:**

CDKN1A was highly expressed in cisplatin-sensitive OC cell lines, and silencing CDKN1A significantly promoted proliferation and cell cycle entry but decreased apoptosis in cisplatin-sensitive OC cells. miR-98-5p targeted CDKN1A to inhibit CDKN1A expression. CAF-derived exosomal miR-98-5p increased OC cell proliferation and cell cycle entry, but suppressed cell apoptosis. Furthermore, exosomal miR-98-5p promoted cisplatin resistance and downregulated CDKN1A in nude mice.

**Conclusion:**

Collectively, CAF-derived exosomes carrying overexpressed miR-98-5p promote cisplatin resistance in OC by downregulating CDKN1A.

## Background

Ovarian cancer (OC) is regarded as the 7th leading cancer diagnosis and the 8th major reason for cancer mortality among global females [1], which is featured by extensive peritoneal dissemination with ascites [2]. OC could be divided into four major histological subtypes, serous, endometrioid, mucinous and clear cell [3]. The risk factors for OC include rare use of oral contraceptive, menstrual and hormonal factors, smoking, dietary factors, the lack of physical activity, and a family history [4]. The recommended treatments for OC include the use of cisplatin as an anti-tumor drug against OC; however, the recurrent tumors post cisplatin therapy become chemoresistant [5]. Moreover, cancer-associated fibroblasts (CAFs), an abundant stromal cell type in various cancers [6], have been reported to cause unsatisfactory prognosis of OC [7]. Notably, CAFs are able to secrete exosomes under the tumor microenvironment, and it has been reviewed that exosomes are implicated in both cancer progression and drug resistance [8].

Exosomes are identified as membrane vesicles of 40–100 nm, which are of endocytic origin secreted by the majority of cell types in vitro [9]. Exosomal microRNAs (miRs) have attracted increasing attention and have been recognized as biomarkers for cancers [10]. As previously reported, miR-98-5p participated in the control of cisplatin resistance in epithelial OC, the expression of which was correlated with unfavorable outcome of epithelial OC patients [11]. Moreover, hsa-miR-98-5p was indicated as a promising target in clinical cisplatin treatment for non-small cell lung cancer [12].

Cyclin-dependent kinase inhibitor 1A (CDKN1A, p21), belonging to the Cip/Kip family, has been identified as a target of anti-cancer drugs [13]. As reported previously, CDKN1A could reverse the promoting function of miR-33b-3p on cisplatin resistance in lung cancer cells [14]. Moreover, another study also documented that increased expression of CDKN1A could promote the sensitivity of OC cells to cisplatin [15]. In our study, the binding site between miR-98-5p and CDKN1A was identified based on the prediction through TargetScan online website. On the basis of the aforementioned results and reports, we proposed the hypothesis that CAF-derived exosomes overexpressing miR-98-5p influenced the cisplatin resistance in OC through targeting CDKN1A. Therefore, CAFs were co-cultured with OC cells to test this hypothesis.

## Materials and methods

### Ethics approval and consent to participate

The present study protocols had been approved by General Hospital of Ningxia Medical University. All participating patients signed informed consents prior to the study. All protocols were conducted based on the ethics statement of the *Declaration of Helsinki*. The animal experiments were carried out strictly following the Guide for the Care and Use of Laboratory Animals of the National Institutes of Health.

### Study subjects

In total, 42 patients with OC (aged 45–72 years; an average age of 53.52 ± 8.08 years old) who underwent surgical resection at the General Hospital of Ningxia Medical University from August 2017 to October 2018 were enrolled in this study. Based on the FIGO 2008 staging criteria, among the 42 patients, 26 patients were in stage I-II and 16 patients in stage III. Thirty normal ovarian tissues were collected as controls from healthy individuals (aged 42–71 years, with an average age of 54.33 ± 6.94 years old) who underwent ovariectomy during the same period. The OC tissues and normal ovarian tissues were fresh and intact and histopathologically confirmed. These tissue were rinsed with phosphate-buffer saline (PBS) containing 20% antibiotics, and then detached with collagenase I (Sigma-Aldrich Chemical Company, St Louis, MO, USA) and hyaluronidase (Sigma-Aldrich Chemical Company, St Louis, MO, USA) to isolate major normal fibroblasts (NFs) and CAFs [16].

### In vitro culture of cells

Human normal ovarian epithelial cell line HOSEpiC and human OC cell lines SKOV3, SKOV3/cisplatin (cisplatin-resistant SKOV3 cells) were purchased from Cell Bank Type Culture Collection of Chinese Academy of Sciences (Shanghai, China), and A2780 and A2780/cisplatin (cisplatin-resistant A2780 cells) were provided by Shanghai Biological Technology Co., Ltd. enzyme research (Shanghai, China). Cells were cultured in Roswell Park Memorial Institute-1640 medium (Invitrogen, Carlsbad, CA, USA) supplemented with 10% fetal bovine serum (FBS; Life Technologies, Carlsbad, CA, USA) and 1% penicillin/streptomycin (Beyotime Biotechnology Co., Shanghai, China). CAFs and NFs were co-cultured with Dulbecco’s modified Eagle’s medium (DMEM)/F12 containing 10% FBS at 37 °C in a 5% CO_2_ incubator (thromo3111, Beisheng Medical Devices Co., Ltd., Jinan, China). The cells were passaged every 3 days [17].

### Immunofluorescence

CAFs and NFs were collected and seeded into 6-well plates coated with polylysine. Following three rinses with preheated 0.01 mol/L PBS, the cells were fixed with 4% paraformaldehyde under room temperature conditions for a 30-min period. After three PBS rinses, the cells were sealed with blocking solution (Beyotime Biotechnology Co., Shanghai, China) at 37 °C for 60 min. Afterwards, the cells were probed with rabbit antibodies against α-smooth muscle actin (α-SMA; ab32575, 1: 200), fibroblast activation protein (FAP; ab53066, 1: 50), and fibroblast-specific protein 1 (FSP1; ab124805, 1: 500) overnight at 4 °C. The antibodies were obtained from Abcam Inc., Cambridge, UK. The following day, further incubation of the cells was conducted with the following secondary antibodies: Alexa Fluor 594 conjugated donkey anti-rabbit (1: 400, A21202) and Alexa Fluor 488 conjugated donkey anti-mouse (1: 400, A21207), for 1 h in dark, which were both purchased from Life Technologies (Carlsbad, CA, USA). Subsequently, the cells were stained with 4′,6-diamidino-2-phenylindole (DAPI; Beyotime Biotechnology Co., Shanghai, China) at room temperature for 5 min, and sealed with anti-fade mounting medium (Beyotime Biotechnology Co., Shanghai, China). Finally, a high-content screening imaging system (Image Xpress Micro 4, Molecular Devices, Sunnyvale, CA, USA) was employed for photography.

#### Isolation and characterization of exosomes

CAFs or NFs were cultured on 6-well plates. When the cell confluence reached 80–90%, the culture medium was removed. The cells were rinsed two times with sterile PBS, followed by a 2-h period of culture in 2 mL serum-free DMEM/F12 medium. Afterwards, the culture medium (CM) was collected. CAFs-CM or NFs-CM were subjected to a 30-mie centrifugation at 2000×*g* at 4 °C. A 0.22 μm membrane was applied to filter the supernatant, followed by ultracentrifugation at 100,000×*g* for 90 min. Subsequently, the precipitations were exosomes to be collected, which were then resuspended in sterilized PBS buffer and centrifuged again for 60 min at 100,000×*g* at 4 °C. Following the removal of the supernatant, another rinse, re-suspension and further precipitation, the precipitations were re-suspended with PBS, filtered using a 0.22 μm membrane, and frozen at -20 °C for subsequent use [18, 19].

The isolated exosomes were fixed first with 2% paraformaldehyde, 2.5% glutaraldehyde, 1% osmic acid for 1.5 h, dehydrated using gradient alcohol, embedded, immersed in epoxy resin overnight, and polymerized sequentially at 35 °C, 45 °C and 60 °C for 24 h. Finally, the exosomes were sliced into ultrathin sections and stained with lead with the morphology observed and photographed under transmission electron microscopy (H-600, Hitachi, Tokyo, Japan).

The exosome suspension was diluted by means of gradual dilution, an appropriate amount of which was then added to a nanoparticle tracer (Malvern Instruments, Malvern, Worcestershire, UK) for detection purpose. The diluted samples whose concentration was detected to fluctuate from (1 − 9) × 10^8^/mL were selected for further use. The appropriate background gray level was selected using the operation software, and the motion track of the particles was recorded. The concentration and particle size distribution of the diluted samples were output. The concentration of exosomes from the original suspension was calculated based on the dilution ratio.

### Western blot assay

Cells were lysed for 30 min using radio immunoprecipitation assay lysis buffer containing phenylmethanesulfonyl fluoride (R0010, Beijing Solarbio Science & Technology Co., Ltd., Beijing, China) on ice, and subjected to a 10-min centrifugation at 12,000 r/min at 4 °C. The total protein concentration was determined using a bicinchoninic acid kit (Pierce, Rockford, IL, USA). Next, 50 μg protein was dissolved in 2× sodium dodecyl sulfate (SDS) buffer and boiled for 5 min. Subsequently, the protein samples underwent 10% SDS–polyacrylamide gel electrophoresis and a transfer onto polyvinylidene fluoride membranes (Merck Millipore, Billerica, MA, USA) by the wet transfer method. The membrane was blocked with 5% skim milk under room temperature conditions for 1 h. An overnight incubation of the membrane was then performed at 4 °C with diluted rabbit antibodies against CD63 (ab118307, 1: 50), CD81 (ab109201, 1: 1000), tumor susceptibility gene 101 (TSG101; ab125011, 1: 1000), CDKN1A (p21) (ab109520, 1: 1000) and glyceraldehyde-3-phosphate dehydrogenase (GAPDH; ab8245, 1: 10,000). Afterwards, the membrane was probed with the horseradish peroxidase-labeled secondary antibody, goat anti-rabbit antibody to immunoglobulin G (IgG; ab205719, 1: 2000) for 1 h. After a TBST rinse, the membrane was developed using enhanced chemiluminescence (BB-3501, (Amersham, Buckinghamshire, UK). Gel imaging system was used for photography, followed by analysis using the Image J software. All the antibodies mentioned were from Abcam Inc. (Cambridge, UK).

### Cell counting kit-8 (CCK-8) assay

OC cells were collected upon reaching logarithmic growth state, and resuspended in order to reach a concentration of 1 × 10^5^ cells/mL. Next, 100 μL cell suspension was subjected to a 24-h incubation at 37 °C with 5% CO_2_ in a 96-well plate. After 24, 48 and 72 h, 10 μL CCK-8 reagent was applied to stain the cells. The optical density (OD) was measured at 450 nm with an enzyme-linked immunometric meter after 24 h.

### Determination of half maximal inhibitory concentration (IC50) of cisplatin

A total of 100 μL cell suspension (1 × 10^5^ cells/mL) was seeded to 96-well plates, followed by treatment with cisplatin at variant concentrations (0, 1, 2, 4, 8 μg/mL; Selleck Chemicals, Houston, TX, USA) for 72 h. The sensitivity of OC cells to cisplatin was then evaluated via the CCK-8 assay, and IC50 was obtained.

### Co-culture of exosomes and OC cells

Exosomes were labeled with PKH67 fluorescent staining solution (Sigma-Aldrich Chemical Company, St Louis, MO, USA). Briefly, 200 μg exosomes and 4 μg PKH67 staining solution were dissolved with 1 mL Dilument C solution (Beyotime, Shanghai, China), respectively, followed by slight mixing for 5 min. After the exosomes underwent a 2-h centrifugation at 100,000 g at 4 °C, the labeled exosomes were collected after another centrifugation at 100,000*g* at 4 °C for 2 h. After A2780 cells were plated to a 24-well plate at a density of 5 × 10^4^ cells each well, they were cultured overnight. The next day, the cells were respectively treated with PBS, CAF-derived exosomes (CAF-exo) or NF-derived exosomes (NF-exo) for 24 h. A Nikon Eclipse Ti confocal laser scanning microscope was used to observe the uptake of exosomes by A2780 cells. Next, the cells subjected to different co-culture were treated with cisplatin for 48 h.

### Cell transfection

A2780 cells were plated into 6-well plates. Upon reaching cell confluency of about 70%, the cells were treated with overexpression-negative control (oe-NC) and oe-CDKN1A plasmids with the use of Lipofectamine 2000 (Invitrogen, Carlsbad, CA, USA). All the plasmids were from Ribobio (Guangzhou, Guangdong, China).

Mimic-NC and miR-98-5p mimic plasmids were transfected into CAFs using the same method described above. After 24 h of transfection, exosomes were isolated from CAFs. Next, the exosomes, including exosomes from CAFs transfected with miR-98-5p or mimic-NC (miR-98-5p-exo or NC-exo) were added into A2780 cells without transfection or A2780 cells after transfection. All the exosomes were preserved for subsequent experimentation.

### Reverse transcription quantitative polymerase chain reaction (RT-qPCR)

Total RNA was collected using a RNeasy MiniKit (Qiagen company, Hilden, Germany). For mRNA and lncRNA, RNA was reversely-transcribed by a reverse transcription kit (RR047A, Takara Bio Inc., Otsu, Shiga, Japan). For miRNA, the obtained RNA was then reversely-transcribed into complementary DNA (cDNA) according to the protocols of the microRNA FirstStrand cDNA Synthesis (Tailing Reaction) kit (B532451-0020, Shanghai Sangon Biotechnology Co. Ltd., Shanghai, China). qPCR was then performed on a real-time fluorescence qPCR detection device (ABI7500, ABI, Foster City, CA, USA) with reference to the SYBR PremixExTaqTMII (Perfect Real Time) kit (DRR081, Takara Bio Inc., Otsu, Shiga, Japan); three duplicated wells were set for each sample. The general negative primers of miRs and the upstream primers of U6 internal reference were extracted from the microRNA FirstStrand DNA synthesis kit. The other primers synthesized by Shanghai Sangon Biotechnology Co., Ltd. (Shanghai, China) are listed in Table 1. Target gene expression was normalized to that of GAPDH, and miR-98-5p expression was normalized to that of U6. Relative expression was calculated using the 2-ΔΔCt method.

**Table 1:** Primer sequences for RT-qPCR

Target gene	Primer sequence (5′–3′)
miR-98-5p	F: GGAAAATCGCCATAGCCAGG
	R: AGATCAGGGTGGCCCCATTT
CDKN1A	F: GGAAGGGACACACAAGAAGAAG
	R: AGCCTCTACTGCCACCATCTTA
GAPDH	F: CCAGGAAATGAGCTTGACAAAGTG
	R: AAGGTCATCCCTGAGCTGAGCTG

RT-qPCR, reverse transcription quantitative polymerase chain reaction; miR-98-5p, microRNA-98-5p; CDKN1A, cyclin-dependent kinase inhibitor 1A; GAPDH, glyceraldehyde-3-phosphate dehydrogenase; F, forward; R, reverse

### Colony formation assay

In total, 2 mL 0.6% bottom agarose (Gibco Company, Grand Island, NY, USA) was supplemented to each well of a 6-well plate. After the agarose at the bottom was coagulated, the cells were evenly suspended in 0.3% agarose at 37 °C. Next, 2 mL cell suspension was immediately added to the 6-well plate (2000 cells/well) and allowed to stand for 10 min at 4 °C. The cells were maintained in an incubator for 14 days at 37 °C. The number of formed clones (≥ 50 cells was regarded as one clone) was counted under a microscope. Three parallel wells were set up to obtain the average value.

### Flow cytometry

Extracted cells were resuspended to adjust the density into 1 × 10^6^ cells/mL. Subsequently, 1 mL cells were fixed overnight at 4 °C using precooled 70% ethanol solution. Following re-suspension, 100 μL cell suspension (no less than 1 × 10^6^ cells/mL) was treated with 1 mL 50 mg/L propidium iodide (PI) staining solution contained RNAase (Sigma-Aldrich Chemical Company, St Louis, MO, USA) for 30 min in darkness. Afterwards, a 300-mesh nylon mesh was used to filter the cell suspension. Finally, cell cycle distribution was detected using a Gallios flow cytometer (Beckman Coulter, Inc., Chaska, MN, USA) at the excitation wavelength of 488 nm.

Annexin V-fluorescein isothiocyanate (FITC)/PI staining was conducted to detect cell apoptosis. The procedures used in cell apoptosis detection were the same with those used in cell cycle detection. The cells were dyed using 10 μL Annexin V-FITC as well as 5 μL PI under room temperature conditions for 15 min in darkness. Finally, cell apoptosis was tested with the use of a flow cytometer.

### Dual luciferase reporter assay

CDKN1A 3′ untranslated region (UTR) gene fragments were artificially synthesized, and then introduced into pMIR-reporter (Beijing Huayueyang Biotechnology, Beijing, China). The mutant form in the potential miR-98-5p binding sites was also constructed. The wild type (WT) and mutant (MUT) luciferase reporter plasmids were co-transfected into HEK293T cells along with miR-98-5p, respectively. The activities of both firefly luciferase (M1) and Ranilla luciferase (M2) were determined using a dual luciferase reporter assay kit (Promega, Madison, WI, USA). The target gene as well as the luciferase activity of gene promoter was expressed by M2/M1, and the average of three wells served as the final result.

### Xenograft tumor in nude mice

Thirty specific-pathogen-free female BALB/c nude mice (aged 4–6 weeks) were from Shanghai SLAC Laboratory Animal Co., Ltd. (Shanghai, China) and reared in a routine manner. A2780 cells were subjected to different culture patterns as follows: (1) 1.5 × 10^6^ A2780 cells were exposed to serum-free medium; (2) 1.5 × 10^6^ A2780 cells were cultured in serum-free medium containing 20 μg agomir-NC-exo; (3) 1.5 × 10^6^ A2780 cells were cultured in serum-free medium containing 20 μg agomir-miR-98-5p-exo. After 12 h, A2780 cells were thoroughly rinsed to remove the remaining exosomes.

The A2780 cells (2 × 10^6^ cells in 25 mL PBS) cultured in different ways were delivered into the groin of the nude mice in a separate manner, in order to develop a subcutaneous transplantation nude mouse model of OC. Subsequently, cisplatin, at a dose of 2.5 mg/kg/2 days, was administrated into the abdominal cavity of the nude mice [20] for a total of 21 times. The length as well as the width of the transplanted tumors in the nude mice was measured every 7 days utilizing a digital caliper. The volume of tumors was calculated by this formula: volume = length × width^2^ × 0.5 [21] to plot the growth curve. After 42 days, the mice were euthanized by excessive anesthesia, and the tumors were removed, and weighed.

### Statistical analysis

Data analysis was conducted utilizing the SPSS 21.0 software (IBM Corp., Armonk, NY, USA). All quantitative data were shown as mean ± standard deviation. Unpaired *t* test was applied to compare the unpaired data between two groups that conformed to normal distribution and homogeneity of variance. One-way analysis of variance (ANOVA) was used to compare data among multiple groups, followed by a Tukey multiple comparisons posttest. Comparisons among multiple groups at various time points were analyzed using repeated measures ANOVA, and Bonferroni post hoc test was further employed. The correlation between two indices was analyzed by means of Pearson’s correlation analysis. *p* < 0.05 was indicative of a statistically significant difference.

## Results

### Silencing CDKN1A promotes cisplatin resistance in cisplatin-sensitive OC cells

**Fig. 1:**
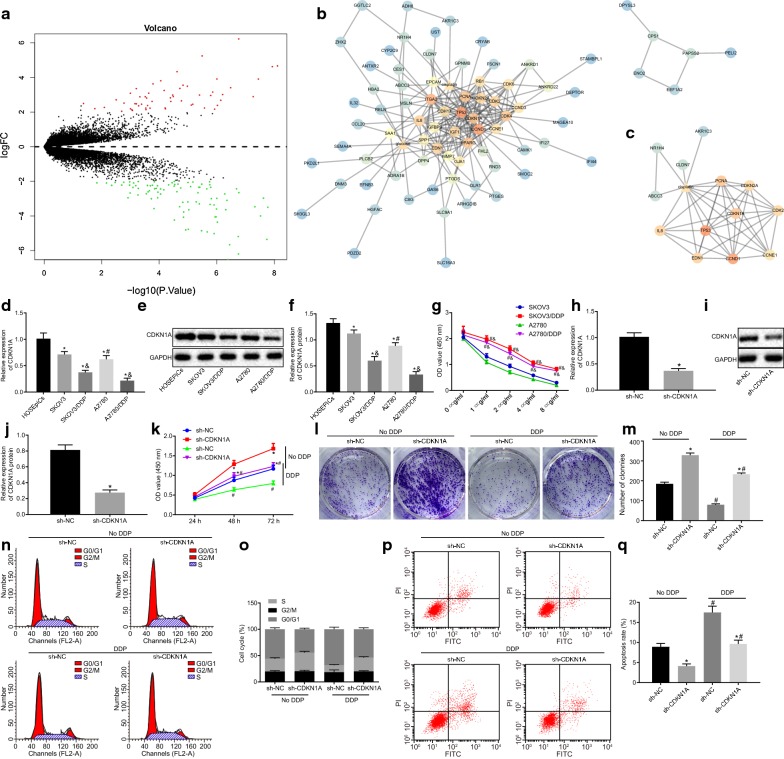
Silence of CDKN1A induces cisplatin resistance in cisplatin-sensitive OC cells. **a** Heatmap of the OC GSE58470 dataset. The X-label indicates the logP value, and the Y-label indicates the logFC value. Each dot indicates one gene, the red dots indicate the genes highly expressed in the drug-resistant samples, and the green dots indicate the genes poorly expressed in the drug-resistant samples. **b** Correlation analysis between differential genes and cisplatin. In the graph, triangles represent chemicals, circles represent genes, and colors represent the core degree of the factors in the network graph. The darker color indicates the higher core degree. **c** Subnetworks of differential genes related to cisplatin in the whole network map. **d** The expression of CDKN1A in OC cell lines and human normal ovarian epithelial cell line as detected by RT-qPCR. **e** The expression of CDKN1A in OC cell lines and human normal ovarian epithelial cell line as detected by Western blot analysis. **f** Statistical analysis for **e**. **g** Detection of IC50 of cisplatin in OC cells. **h** The expression of CDKN1A in OC cells after silencing CDKN1A as detected by RT-qPCR. I, The expression of CDKN1A in OC cells after silencing CDKN1A as detected by Western blot analysis. J, Statistical analysis for **i**. **k** OC cell proliferation after silencing CDKN1A as detected by CCK-8 assay. **l** Colony formation ability after silencing CDKN1A as detected by colony formation assay. **m** Statistical analysis for **l**. **n** OC cell cycle distribution after silencing CDKN1A as detected by flow cytometry. **o** Statistical analysis for **n**. **p** OC cell apoptosis after silencing CDKN1A as detected by flow cytometry. **q** Statistical analysis for **p**. *p < 0.05 vs. HOSEpiCs cell line or sh-NC; ^#^*p* < 0.05 vs. SKOV3 cell line or cells without treatment of cisplatin; ^&^*p* < 0.05 vs. cisplatin-sensitive OC cell lines. These data were measurement data, expressed as mean ± standard deviation. Data between two groups were compared using unpaired *t*-test. Data among multiple groups were compared using one-way analysis of variance and further analyzed with Tukey’s post hoc test. Comparisons among multiple groups at different time points were analyzed using repeated measures ANOVA, followed by Bonferroni post hoc test. The experiment was repeated three times

The cisplatin-treated OC dataset, GSE58470, was downloaded from the Gene Expression Omnibus (GEO) database (https://www.ncbi.nlm.nih.gov/geo/), which included both cisplatin-sensitive OC data and cisplatin-resistant OC data. With |logFC| > 2 and a *p* value < 0.05 as screening criteria, 144 differentially expressed genes were obtained (Fig. 1a). Furthermore, the correlation between the 144 differential genes and cisplatin was analyzed on the STITCH database (http://stitch.embl.de/), and the drug-gene interaction network was constructed (Fig. 1b). The network of cisplatin-related genes were separately constructed (Fig. 1c). It was detected that there existed a direct relationship of TP53 and CDKN1A with cisplatin. Furthermore, the expression pattern of the top 4 genes in regard to core degree (PCNA, TP53, CDKN1A, CCND1) in cisplatin-resistant OC cells was determined, which demonstrated that CDKN1A expression exhibited the most significant change. In addition, RT-qPCR and Western blot analysis displayed that the expression patterns of CDKN1A in OC cell lines were as follows: HOSEpiCs > SKOV3 > A2780 > SKOV3/cisplatin > A2780/cisplatin (Fig. 1D-F), indicating that higher CDKN1A expression in cisplatin-sensitive OC cell lines as well as lower CDKN1A expression in cisplatin-resistant OC cell lines. Moreover, the IC50 of cisplatin on SKOV3, SKOV3/cisplatin, A2780 and A2780/cisplatin cell lines was detected to be 2.43 ± 0.14, 6.52 ± 0.17, 2.51 ± 0.17 and 6.65 ± 0.26 μg/mL, respectively (Fig. 1g). Therefore, in this study, the A2780 cell line at a cisplatin IC50 of 2.5 μg/mL was designated for the following experimentation.

The expression of CDKN1A was then silenced in A2780 cells. RT-qPCR and Western blot analysis showed that CDKN1A was significantly down-regulated after transfection of sh-CDKN1A (*p* < 0.05) (Fig. 1h–j). CCK-8 assay and colony formation assay illustrated that in both cisplatin-treated cells and cells without cisplatin treatment, cell proliferation and colony formation ability were notably enhanced after silencing CDKN1A treatment (*p* < 0.05). However, after cisplatin treatment, cell proliferation and the number of clones were progressively diminished in A2780 cells, which could be partially rescued after silencing CDKN1A (*p* < 0.05, Fig. 1k–m). As demonstrated by flow cytometry, in both cisplatin-treated cells and cells without cisplatin treatment, cell apoptotic rate in addition to the proportion of cells in the G1 phase displayed an obvious decline while the proportion of cells in the S phase had a marked elevation in the presence of silencing CDKN1A (*p* < 0.05). However, following cisplatin treatment, cell apoptotic rate and the proportion of cells in the G1 phase were obviously increased, while the proportion of cells in the S phase was markedly decreased, which could be partially blocked after silence of CDKN1A (all *p* < 0.05, Fig. 1n–q). These results suggested that silencing CDKN1A could significantly increase proliferation and decrease apoptosis of cisplatin-sensitive OC cells.

### MiR-98-5p targets at and inhibits CDKN1A

**Fig. 2:**
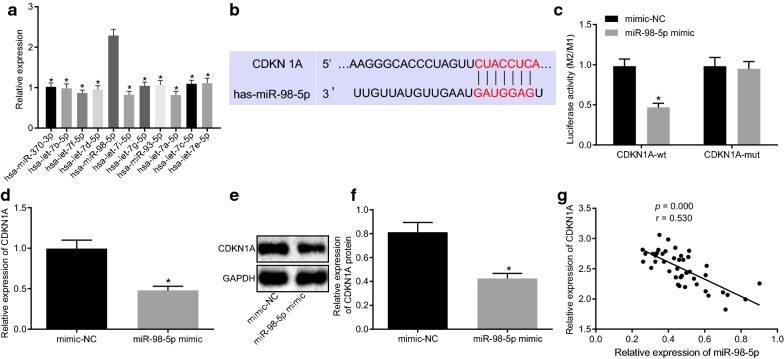
miR-98-5p targets CDKN1A to inhibit CDKN1A expression. **a** The expression of related miRs in the CAF-derived exosomes as detected by RT-qPCR. **b** The binding sites between CDKN1A and miR-98-5p predicted by the TargetScan website. **c** The binding of miR-98-5p to CDKN1A as verified by dual luciferase reporter assay. **d** The expression of CDKN1A in OC cells after treatment of miR-98-5p mimic as detected by RT-qPCR. **e** The expression of CDKN1A in OC cells after treatment of miR-98-5p mimic as detected by Western blot analysis. **f** Statistical analysis for **e**. **g** Pearson’s correlation analysis of the correlation between the expression of miR-98-5p and CDKN1A in OC cells. *p < 0.05 vs. has-miR-98-5p or the treatment of mimic-NC. These data were measurement data, expressed as mean ± standard deviation. Data between two groups were compared using unpaired *t*-test. Data among multiple groups were compared using one-way analysis of variance and further analyzed with Tukey’s post hoc test. The correlation between two indices was analyzed using Pearson’s correlation analysis. The experiment was repeated three times

Among the miRs in CAF-derived exosomes, miR-98-5p was observed to show a high expression in the CAF-derived exosomes (Fig. 2a). Thus, we decided to explore the relationship between miR-98-5p and CDKN1A.

It was predicted that there was a binding site between miR-98-5p and CDKN1A based on TargetScan online website (Fig. 2b). As revealed by dual luciferase reporter assay, a significant decrease was displayed regarding the luciferase activity of CDKN1A-WT in response to miR-98-5p mimic (*p* < 0.05), whereas the luciferase activity of CDKN1A-MUT remained significantly unchanged by miR-98-5p mimic (*p* > 0.05; Fig. 2c). As illustrated by RT-qPCR and Western blot analysis in Fig. 2d–f, the CDKN1A expression was remarkably reduced by miR-98-5p mimic. Pearson’s correlation analysis showed that in A2780 cells, the miR-98-5p expression shared negative correlation with the expression of CDKN1A (r = -5.517, *p* < 0.05; Fig. 2g). Therefore, it was concluded that CDKN1A was the target gene of miR-98-5p.

### CAF-derived exosomes overexpress miR-98-5p and promote cisplatin resistance in OC cells

**Fig. 3:**
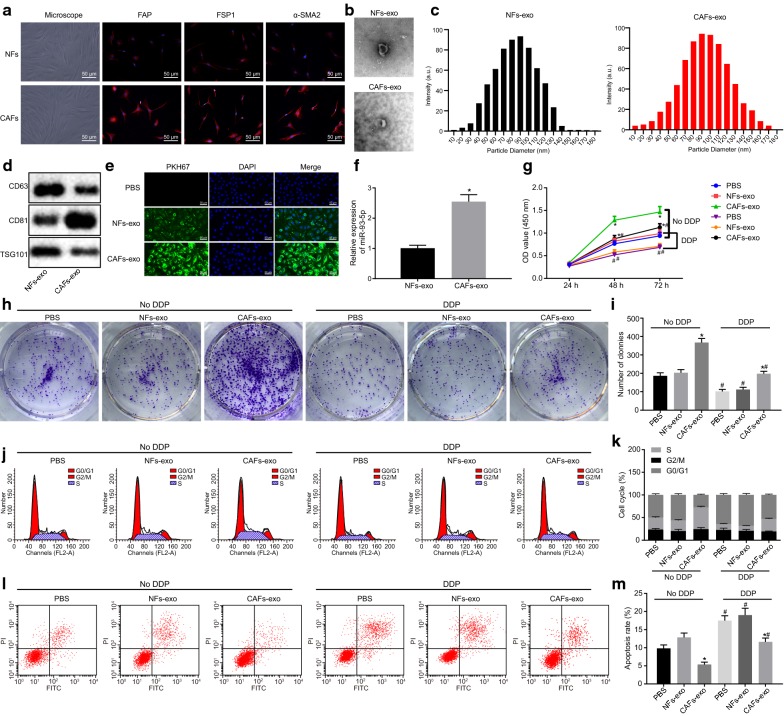
CAF-derived exosomes promote cisplatin resistance in OC cells. **a** Morphology of fibroblasts based on the observation under the microscope and the expression of specific marker proteins (α-SMA, FAP and FSP1) in CAFs as detected by immunofluorescence staining (×200). **b** Ultrastructure of the exosomes under transmission electron microscopy (×5000). **c** Particle size of the isolated exosomes as detected by nanotrace analysis. **d** The expression of the exosome surface marker proteins, CD63, CD81 and TSG101, as detected by Western blot analysis. **e** The uptake of exosomes by A2780 cells observed under the laser confocal microscope (× 200). **f** The expression of miR-98-5p in A2780 cells co-cultured with exosomes as detected by RT-qPCR. **g** OC cell proliferation after co-culture with exosomes and treatment of cisplatin as detected by CCK-8 assay. **h** OC colony formation ability after co-culture with exosomes and treatment of cisplatin as detected by colony formation assay. I, Statistical analysis for **h**. **j** OC cell cycle distribution after co-culture with exosomes and treatment of cisplatin as detected by flow cytometry. **k** Statistical analysis for **j**. **l** OC cell apoptosis after co-culture with exosomes and treatment of cisplatin as detected by flow cytometry. **m** Statistical analysis for **l**. *p < 0.05 vs. PBS; ^#^*p* < 0.05 vs. cells without treatment of cisplatin. These data were measurement data, expressed as mean ± standard deviation. Data between two groups were compared using unpaired *t*-test. Data among multiple groups were compared using one-way analysis of variance and further analyzed with Tukey’s post hoc test. Comparisons among multiple groups at different time points were analyzed using repeated measures ANOVA, followed by Bonferroni post hoc test. The experiment was repeated three times

Based on the observation under the microscope, CAFs and NFs were slender and fibrous. Immunofluorescence exhibited that the expression of specific marker proteins (α-SMA, FAP and FSP1) was enhanced in CAFs (Fig. 3a). Next, the exosomes were isolated from CAFs and NFs. Under the transmission electron microscopy, it was found that the isolated vesicles were round or elliptical membrane vesicles in disk-like structure, with complete capsule (Fig. 3b). Nanotrace analysis showed that the isolated vesicles were structures with an average particle size of 50-100 nm (Fig. 3c). Western blot analysis results exhibited that the isolated exosomes were positive for exosomal markers (CD63, CD81 and TSG101) (Fig. 3d). The above results demonstrated that the exosomes had been successfully isolated.

The fluorescence labeled exosomes from CAFs and NFs were co-cultured with A2780 cells. Through the observation under the laser confocal microscope, it was shown that obvious green fluorescence was observed in the cytoplasm of A2780 cells co-cultured with CAF-exo and NF-exo, with no significant difference in fluorescence intensity between co-cultured with CAF-exo and NF-exo (*p* > 0.05; Fig. 3e). Besides, based on RT-qPCR, the miR-98-5p expression in A2780 cells co-cultured with CAF-exo was profoundly higher than in A2780 cells co-cultured with NF-exo (*p* < 0.05; Fig. 3f).

A2780 cells were co-cultured with exosomes and treated with cisplatin. The results obtained from CCK-8 and colony formation assays displayed that cisplatin treatment significantly reduced the cell proliferation and the number of clones (*p* < 0.05). Relative to PBS treatment, NF-exo did not bring up significant changes in terms of the cell proliferation and the number of clones (all *p* > 0.05), while CAF-exo led to marked increases in the cell proliferation and the number of clones (*p *< 0.05; Fig. 3g–i).

Furthermore, based on the results of flow cytometry, after cisplatin treatment, a notable elevation was found regarding the percentage of cells in the G0/G1 phase, while the percentage of cells in the S phase was markedly reduced, and the apoptotic rate showed a notably increase (*p* < 0.05). Moreover, NF-exo resulted in no significant changes in terms of all the above indicators when compared with PBS treatment (*p* > 0.05). Furthermore, CAF-exo contributed to a marked decrease in the percentage of cells distributed in the G0/G1 phase, a notable elevation in the percentage of cells arrested in the S phase, as along with a decreased apoptotic rate (*p* < 0.05; Fig. 3j–m). These results suggested that CAF-derived exosomes could significantly promote cisplatin resistance in OC cells.

### CAF-derived exosomes promote cisplatin resistance in OC cells by transferring miR-98-5p

**Fig. 4:**
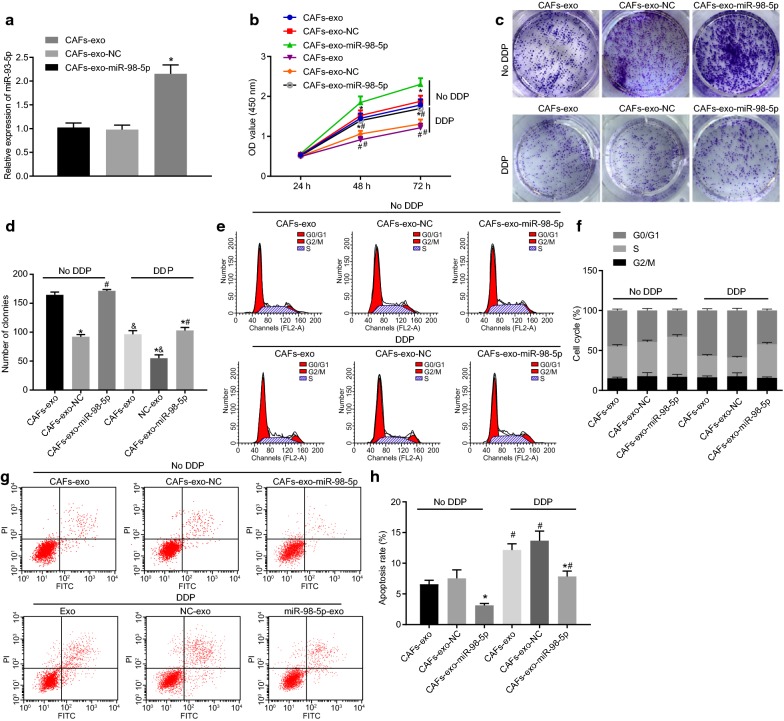
CAFs derive exosomes to deliver miR-98-5p to promote cisplatin resistance in OC cells. **a** The expression of miR-98-5p in exosomes as detected by RT-qPCR. **b** OC cell proliferation after co-culture with exosomes and treatment of cisplatin as detected by CCK-8 assay. **c** OC cell colony formation ability after co-culture with exosomes and treatment of cisplatin as detected by colony formation assay (×200). **d** Statistical analysis for **c**. **e** OC cell cycle distribution after co-culture with exosomes and treatment of cisplatin as detected by flow cytometry. **f** Statistical analysis for **e**. **g** OC cell apoptosis after co-culture with exosomes and treatment of cisplatin as detected by flow cytometry. **h** Statistical analysis for **g** *p < 0.05 vs. NC-exo; ^#^*p* < 0.05 vs. cells without treatment of cisplatin. These data were measurement data, expressed as mean ± standard deviation. Data between two groups were compared using unpaired *t*-test. Data among multiple groups were compared using one-way analysis of variance and further analyzed with Tukey’s post hoc test. Comparisons among multiple groups at different time points were analyzed using repeated measures ANOVA, followed by Bonferroni post hoc test. The experiment was repeated three times

Exosomes were isolated from CAFs after transfection of NC-mimic (NC-exo) and miR-98-5p-mimic (miR-98-5p-exo). As illustrated in RT-qPCR, no significant difference existed in terms of miR-98-5p expression between NC-exo and exosomes from CAFs without any transfection (exo) (*p* > 0.05). However, miR-98-5p expression was increased significantly in miR-98-5p-exo (Fig. 4a). Subsequently, the exosomes were co-cultured with A2780 cells and then treated with cisplatin. Based on the results from CCK-8 and colony formation assays, treatment with cisplatin contributed to significant declines in the cell proliferation and the number of clones (*p* < 0.05). No significant changes were detected with regard to the cell proliferation and the number of clones between A2780 cells co-cultured with NC-exo and exo (*p* > 0.05). However, the A2780 cell proliferation and the number of clones displayed an obviously promotion following miR-98-5p-exo treatment (*p* < 0.05) (Fig. 4b–d).

As displayed in flow cytometry, following treatment with cisplatin, a notable increase was detected regarding the proportion of cells in the G0/G1 phase, while the proportion of cells in the S phase showed a decline, along with an elevated apoptotic rate in A2780 cells (*p* < 0.05). Moreover, co-culture with NC-exo resulted in no significant changes in terms of all the above indicators when compared with co-culture with exo (*p* > 0.05). Furthermore, miR-98-5p-exo resulted in diminished proportion of cells distributed in the G0/G1 phase, increased proportion of cells arrested in the S phase, as addition to decreased apoptotic rate (*p* < 0.05) (Fig. 4e–h). Collectively, it was suggested that CAF-derived exosomes overexpressing miR-98-5p could promote cisplatin resistance in OC.

### CAFs transfer exosomal miR-98-5p to promote cisplatin resistance in OC cells by targeting CDKN1A

**Fig. 5:**
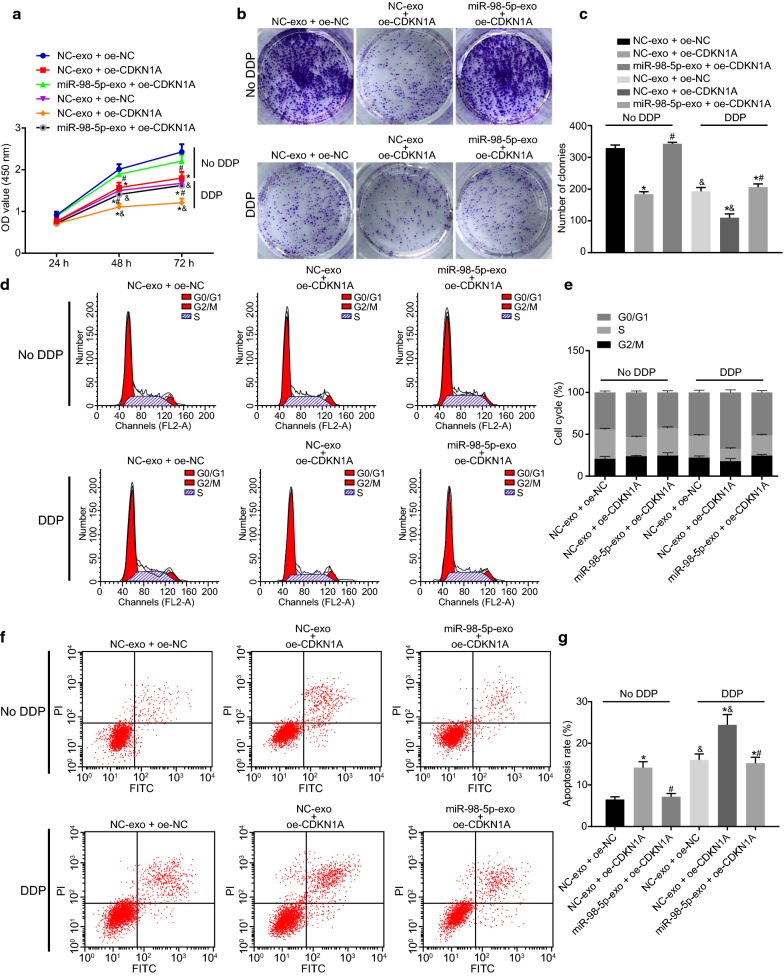
CAF-derived exosomal miR-98-5p stimulates cisplatin resistance in OC cells by targeting CDKN1A. A2780 cells transfected with oe-CDKN1A or oe-NC were co-cultured with CAF-exo and further treated with cisplatin. **a** Cell proliferation as detected by CCK-8 assay. **b** OC cell colony formation ability as detected by colony formation assay. **c** Statistical analysis for **b**. **d** OC cell cycle distribution as detected by flow cytometry. **e** Statistical analysis for **d**. **f** OC cell apoptosis as detected by flow cytometry. **g** Statistical analysis for **f**. *p < 0.05 vs. NC-exo + oe-NC; ^#^*p* < 0.05 vs. NC-exo + oe-CDKN1A; ^&^cells without treatment of cisplatin. These data were measurement data, expressed as mean ± standard deviation. Data between two groups were compared using unpaired *t*-test. Data among multiple groups were compared using one-way analysis of variance and further analyzed with Tukey’s post hoc test. Comparisons among multiple groups at different time points were analyzed using repeated measures ANOVA, followed by Bonferroni post hoc test. The experiment was repeated three times

Subsequently, we further investigated the mechanisms regarding the role that CAF-derived exosomes overexpressing miR-98-5p played in cisplatin resistance in OC cells. The CAF-exo was co-cultured with A2780 cells overexpressing CDKN1A, which were further treated with cisplatin. CCK-8 and colony formation assays documented that cell proliferation and the number of clones were significantly reduced in OC cells after treatment with cisplatin. Overexpression of CDKN1A led to profoundly reduced cell proliferation and the number of clones, which was antagonized by co-culture with miR-98-5p-exo (Fig. 5a–c). As detected in flow cytometry, the proportion of cells in the G0/G1 phase had a marked increase, while the proportion of cells in the S phase was decreased significantly, along with an elevated apoptotic rate in OC cells in the presence of cisplatin. Moreover, following CDKN1A overexpression, the proportion of cells in the G0/G1 phase reduced, while that of cells in the S phase increased, plus a decreased apoptotic rate in OC cells, which was abrogated by the combination of miR-98-5p-exo and oe-CDKN1A (Fig. 5d–g). As expected, CAF-derived exosomes could transfer miR-98-5p to facilitate OC cells resistance to cisplatin through targeting CDKN1A.

### Exosomal miR-98-5p inhibits CDKN1A  expression and promotes cisplatin resistance in transplanted tumors in nude mice

**Fig. 6:**
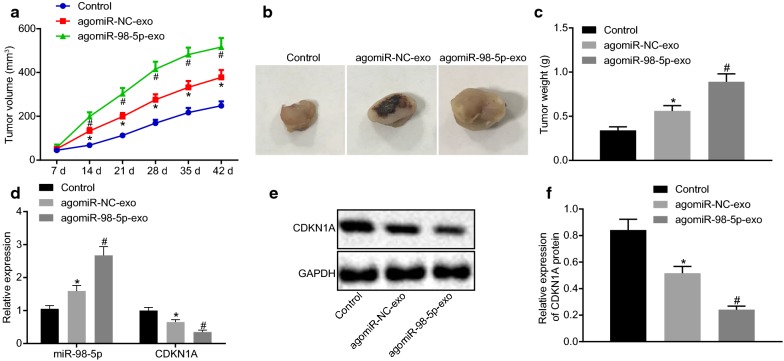
Exosomal miR-98-5p downregulates CDKN1A and promotes cisplatin resistance in transplanted tumors in nude mice. Nude mice were injected with agomir-NC-exo and agomir-miR-98-5p-exo. **a** The volume curve of transplanted tumors in nude mice. **b** observation of tumors from nude mice. **c** The weight of transplanted tumors in nude mice. **d** miR-98-5p and CDKN1A expression in tumor tissues as detected by RT-qPCR. **e** CDKN1A expression as detected by Western blot analysis. **f** Statistical analysis for **e**. *p < 0.05 vs. control mice; ^#^*p* < 0.05 vs. the injection of agomir-NC-exo. These data were measurement data, expressed as mean ± standard deviation. Data between two groups were compared using unpaired *t*-test. Data among multiple groups were compared using one-way analysis of variance and further analyzed with Tukey’s post hoc test. Comparisons among multiple groups at different time points were analyzed using repeated measures ANOVA, followed by Bonferroni post hoc test. The experiment was repeated three times

In order to investigate whether the CAF-derived exosomes could deliver miR-98-5p to influence the cisplatin resistance in OC via CDKN1A in vivo, we injected cisplatin into the abdominal cavity of nude mice transplanted with subcutaneous tumors. It was displayed that the volume as well as weight of tumors in the mice injected with agomir-NC-exo was notably increased relative to control mice (all *p* < 0.05). Moreover, the mice injected with agomir-miR-98-5p-exo had increased tumor volume and weight (Fig. 6a–c). In addition, RT-qPCR and Western blot assays were applied to assess miR-98-5p and CDKN1A expression in tumors of nude mice. miR-98-5p expression was markedly increased, while CDKN1A expression was notably decreased in the nude mice injected with agomir-NC-exo versus control mice (all *p* < 0.05). Agomir-miR-98-5p-exo resulted in further increase in miR-98-5p expression and decrease in CDKN1A expression (Fig. 6d–f). In conclusion, exosomal miR-98-5p could promote cisplatin resistance and downregulate CDKN1A in the xenograft model of OC.

## Discussion

OC is a gynecological tumor with a high mortality rate, whose first-line therapy is combination of radiation treatment and chemotherapy [22]. However, drug resistance is one of the reasons that can lead to therapy failure. The crucial role of exosomes and “exosomal shuttle miR” in both tumorigenesis and drug resistance has been highlighted previously [23]. In this study, the main aim was to examine the functions of CAF-derived exosomes overexpressing miR-98-5p on cisplatin resistance in OC. The findings demonstrated that CAF-derived exosomes could deliver miR-98-5p to facilitate cisplatin resistance in OC, which was achieved by regulating CDKN1A.

Initially, the present study provided evidence to demonstrate that the expression of CDKN1A was higher in cisplatin-sensitive OC cell lines than that in cisplatin-resistant OC cell lines. CDKN1A is defined as a vital regulator for cell cycle arrest and apoptosis [24]. It was found that CDKN1A aided in proliferation restriction of OC cells [25]. Moreover, downregulation of CDKN1A was also found to be accountable for OC cell growth [26]. It has been documented that CDKN1A is a biomarker for response to systemic adjuvant therapies and is associated with drug resistance in human tumors [27]. In line with our study, the sensitization of cisplatin in OC cells by kaempferol was found to be in part due to the upregulation of CDKN1A [28]. Furthermore, in the current study, miR-98-5p was predicted based on the TargetScan website to be the upstream regulatory miRNA of CDKN1A, which was verified by dual luciferase reporter assay. In fact, miR-98-5p has been discovered to participate in cisplatin resistance. For instance, miR-98-5p, by targeting Dicer1, is able to cause cisplatin resistance in epithelial OC cells through the suppression of miR-152 biogenesis [11]. Moreover, in a previous study, downregulated hsa-miR-98-5p was found to potentiate the efficacy of cisplatin in non-small cell lung cancer A549 cells [12].

Another important finding uncovered in this study was that the expression of miR-98-5p in A2780 cells treated with CAF-exo was notably higher than in A2780 cells treated with NF-exo. It was further demonstrated that CAF-derived exosomes could lead to a significant promotion in cisplatin resistance in OC cells. Exosomes can be taken up by other cells, thereby being able to transfer biological messages to nearby cells, and this cell-to-cell communication can affect pathogenesis of some diseases, such as human cancers [29]. It is worthy to note that CAFs can secrete paracrine factors including exosomes and thus regulate proliferation, invasion and cell signaling of cancer cells [30]. It has been discovered that tumor cells can communicate with CAFs via exosomes, thereby constructing a bidirectional cross talk to facilitate both tumor growth, and drug resistance [31]. A previous study on OC cell also showed that fibroblasts-derived exosomes carrying TGFβ1 were capable of promoting epithelial-mesenchymal transition of OC cells [32]. Similar with our results, CAF-derived exosomes were also reported to result in elevation of chemoresistance-inducing factor, Snail, in recipient pancreatic cancer epithelial cells and promote proliferation as well as drug resistance in pancreatic cancer [33].

Furthermore, we demonstrated the promoting role of CAF-derived exosomes in cisplatin resistance in OC, as reflected by enhanced OC cell proliferation and colony formation, and inhibited cell apoptosis, which was mediated by overexpression of miR-98-5p. miRs have been found in exosomes, which are capable of transferring miRs and serving as delivery vehicles for therapeutic molecules [34, 35]. Similarly, highly expressed miR-98-5p was found in Parp1-deficient embryonic stem cell-derived exosomes [36]. Supportably, multiple exosomal miRs have been reported to be implicated in cancer progression or drug resistance. For instance, transfer of stroma-derived miR-21 via exosomes could induce paclitaxel resistance in OC cells by targeting APAF1 [37]. Similar with the finding obtained in this study, exosomal miRs released by CAFs could accelerate the progression of an aggressive breast cancer cell phenotype [38]. Thus, it was concluded that CAF-derived exosomes delivered miR-98-5p to downregulate CDKN1A, thereby promoting OC cells resistance to cisplatin.

## Conclusion

**Fig. 7:**
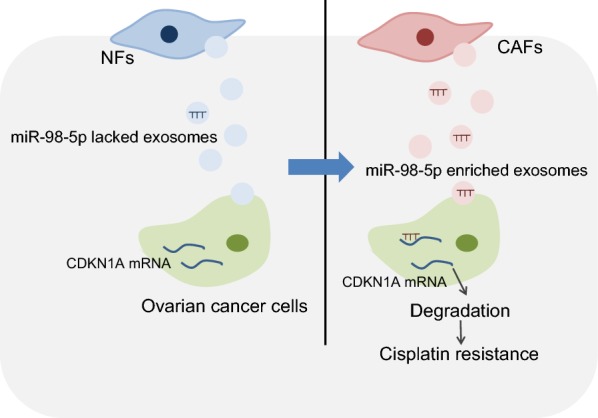
The mechanism of CAF-released exosomal miR-98-5p on OC cell cisplatin resistance via CDKN1A. CAF-derived exosomal miR-98-5p downregulates CDKN1A and promotes cisplatin resistance in OC cells

In summary, the results of this study demonstrated that CAFs could secrete exosomes to deliver miR-98-5p, thereby facilitating OC cells resistance to cisplatin through the downregulation of CDKN1A (Fig. 7). Our study suggested that CAF-derived exosomal miR-98-5p can serve as a novel treatment target for OC.

## Data Availability

The datasets generated/analysed during the current study are available.

## Abbreviations

OC: ovarian cancer
CAFs: cancer-associated fibroblasts
miRs: microRNAs
CDKN1A: p21, Cyclin-dependent kinase inhibitor 1A
PBS: phosphate-buffer saline
NFs: normal fibroblasts
DMEM: Dulbecco’s modified Eagle’s medium
CM: culture medium
SDS: sodium dodecyl sulfate
α-SMA: α-smooth muscle actin
FAP: fibroblast activation protein
DAPI: 4′,6-diamidino-2-phenylindole
FSP1: fibroblast-specific protein 1
GAPDH: glyceraldehyde-3-phosphate dehydrogenase
IgG: immunoglobulin G
OD: optical density
CAF-exo: CAF-derived exosomes
NF-exo: NF-derived exosomes
oe-NC: overexpression-negative control
RT-qPCR: reverse transcription quantitative polymerase chain reaction
CCK-8: cell counting kit-8
IC50: half maximal inhibitory concentration
cDNA: complementary DNA
PI: propidium iodide
FITC: fluorescein isothiocyanate
UTR: untranslated region
TUNEL: terminal deoxynucleotidyl transferase-mediated dUTP-biotin nick end labeling
WT: wild type
MUT: mutant
ANOVA: analysis of variance
GEO: Gene Expression Omnibus
